# Association of Positive Affect Instability With All-Cause Mortality in Older Adults in England

**DOI:** 10.1001/jamanetworkopen.2020.7725

**Published:** 2020-07-08

**Authors:** Anthony D. Ong, Andrew Steptoe

**Affiliations:** 1 Department of Human Development, Cornell University, Ithaca, New York; 2 Division of Geriatrics and Palliative Medicine, Weill Cornell Medical College, New York, New York; 3 Department of Behavioural Science and Health, University College London, London, United Kingdom

## Abstract

**Importance:**

There is increasing recognition that positive affective states have a protective association for all-cause mortality. However, positive states of happiness and excitement vary over time, and little is known about the association of fluctuations in positive affect with survival.

**Objective:**

To investigate the association of positive affect instability, conceptualized as fluctuations in momentary positive affect, with mortality in a population-based sample of older adults in England.

**Design, Setting, and Participants:**

This survey study used data from a longitudinal survey collected in wave 2 (2004) of the English Longitudinal Study of Ageing, a nationally representative sample of older men and women living in England, with follow-up continuing until March 2018. Participants included people aged 50 years or older at recruitment. Data were analyzed from September 2019 to April 2020.

**Main Outcomes and Measures:**

The main outcome was all-cause mortality. Positive and negative affect were measured at 4 time points over the course of 1 day: soon after waking, 30 minutes after waking, at 7:00 pm, and at bedtime.

**Results:**

Data were analyzed from 3834 participants (mean [SD] age at baseline, 64.0 [7.4] years; 2082 [54.3%] women) with a mean (SD) follow-up of 12.25 (2.60) years. Adjusting for demographic characteristics, baseline illness, health behaviors, and mean level and instability in negative affect, Cox proportional hazards regression showed that high positive affect instability was associated with greater mortality, with a hazard ratio of 1.25 (95% CI, 1.04-1.49; *P* = .02). Associations did not differ by age, suggesting that the increased mortality risk associated with high positive affect instability was not restricted to older ages.

**Conclusion and Relevance:**

These findings suggest that temporal fluctuations in positive affect were associated with mortality risk in older adults. These findings illustrate the value of incorporating dynamic assessments of positive affect in distal health outcomes such as mortality.

## Key Points

**Question:**

Are temporal fluctuations in positive affect associated with mortality risk in older adults?

**Findings:**

In this survey study of 3834 adults aged 50 years or older, greater instability of momentary positive affect was associated with increased risk of mortality.

**Meaning:**

This finding suggests that instability of positive affective states in everyday life is relevant to health in old age.

## Teaser

This survey study examines the association of instability of positive affect with all-cause mortality in older adults in England.

## Introduction

Growing evidence suggests that positive affect is positively associated with restorative health behaviors and favorable biological function^1,2,3,4^ and inversely associated with illness morbidity and disease risk.^5,6,7,8^ Meta-analytical reviews have found that high levels of positive affect are associated with longer survival, independent of comorbidities.^9,10,11,12^ The maintenance of positive affect may be particularly important at older ages when the accrual of physiological deficits increases risk of disease and premature mortality.^13,14^

To our knowledge, studies analyzing the associations of positive affect with mortality have focused exclusively on the mean level of positive feelings.^15,16^ However, positive affect fluctuates within individuals over the short and long term, and this intraindividual variability contains added value over mean levels of positive affect in the association of psychological well-being.^17,18,19^ For example, day-to-day variability (ie, ups and downs) in positive affect has been associated with more depressive and somatic symptoms^20,21^ and poor sleep outcomes.^22,23^ The biological correlates of positive affect intraindividual variability have also received attention, with several studies showing protective associations of low positive affect variability with daily cortisol profiles,^24^ immunocompetence,^25^ and heart rate variability.^26^ Thus, the examination of affect dynamics in everyday life may offer additional information that is not captured in traditional global reports assessed at 1 point in time.

A key question is whether temporal fluctuations in positive affect are informative of mortality risk above and beyond mean levels of positive affect. Ong and Ram^17^ characterized an individual’s positive affect as either enduring or fragile. While positive affect that is enduring reflects mean levels that are relatively stable, positive affect that is fragile reflects short-term fluctuations over time. In this study, we focus on 1 marker of fragility, affective instability. Unlike affective variability indices (eg, SD) that reflect how much affect deviates around its mean, affective instability indices capture the variability and temporal dependency of affective changes.^27^ One such index, the mean squared successive difference (MSSD), has been preferred among recent ecological momentary assessment (EMA) studies of negative affect dysregulation in clinical populations.^27,28,29^ These studies suggest that, in addition to displaying higher variability, individuals with various psychiatric disorders (eg, borderline personality, major depressive, posttraumatic stress) experience more frequent fluctuations in negative affect, quantified by relatively low temporal dependency.

To date, it remains unclear what implications elevated instability in positive affect states may hold for recognizing risk among healthy individuals. Examining stable and dynamic aspects of positive affect highlights the clinical and public health importance of further investigating the salutary health outcomes associated with positive affect that are distinct from the outcomes associated with low negative affect.^13,17,30^ Accordingly, we examine associations of individuals’ experienced affective instability and mean affect, as derived from EMA reports, with mortality in a large longitudinal cohort study of adults aged 50 years or older. Fluctuations in positive affect were assessed by means of EMA, a standard ambulatory assessment tool for capturing affective responses and experience as they unfold in real time.^3,15,31^ We hypothesized that greater positive affect instability would be associated with increased risk of mortality, independently of positive affect mean level, baseline illness, or other covariates. A second aim of this analysis was to explore whether associations of positive affect instability with mortality were independent of mean level and instability in negative affective states.

## Methods

### Study Population

Data were analyzed from the English Longitudinal Study of Ageing, a longitudinal population study of noninstitutionalized men and women aged 50 years or older living in England.^32^ As part of wave 2 in 2004, a subsample of participants aged 52 to 79 years completed 4 EMA assessments during a single day. Participant follow-up continued until March 2018. Survey response rates are based on the American Association for Public Opinion Research (AAPOR) reporting guideline. They have been calculated from a number of sources, including outcome codes from fieldwork, sampling recontact information, and mortality updates. We excluded individuals with cancer at baseline and individuals with missing data on 1 or more covariates. The study was approved through the National Research Ethics Service, and all participants provided verbal informed consent.

### Measures

Mortality data were obtained through linkage with the National Health Service Central Data Registry from baseline until March 2018. Positive and negative affects were measured at 4 time points over the course of 1 day: soon after waking, 30 minutes after waking, at 7:00 pm, and at bedtime. Participants were asked to rate the extent to which they felt happy, excited, content, worried, anxious, and fearful on 4-point scales ranging from 0, indicating not at all, to 3, indicating extremely. Positive affect was computed by calculating the mean of 3 positive states (ie, happy, excited, and content), and negative affect by calculating the mean of 3 negative states (ie, worried, anxious, and fearful). The internal consistency for positive and negative affect measures was good (Cronbach α values: positive affect, 0.90; negative affect, 0.92). Mean positive and negative affects were computed based on the ratings from the 4 time points, with higher scores indicating greater affect. Positive and negative affect instabilities were calculated using MSSD, which captures the extent of individuals’ change from 1 point to the next and therefore provides a measure of variability (ie, mean magnitude of affective changes) and temporal dependency (ie, mean frequency of affective changes). High values of MSSD correspond to high variability and low temporal dependency of scores. The limitations of variability and temporal dependency as standalone indicators of affect dynamics have been illustrated by Jahng et al^27^ who present simulated time series showing that 2 individuals with the same variance can have different values of MSSD. The squared difference between successive observations at occasions *i* + 1 and *i* for a time series of *n* measurement occasions is given by
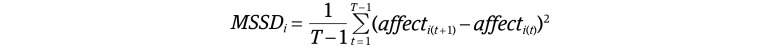
This index was calculated separately for positive affect and negative affect.

Information was available about age, sex, race/ethnicity (classified as white or other), paid employment, and marital status. Education was based on the highest qualifications obtained and divided into 3 categories: low (ie, no qualifications), intermediate (ie, junior and senior high school), and higher (ie, college). Social class was based on the National Statistics Socio-economic Classification, and was divided into routine, intermediate, and managerial.^33^ Baseline illness was assessed with information about diagnosis of coronary heart disease, stroke, diabetes, or chronic lung disease. To assess other long-term conditions that are not included among these formal diagnoses, participants indicated whether they suffered from limiting long-standing conditions.^34^ Current smoking, participation in moderate to vigorous physical activity at least once per week, and drinking alcohol at least daily were also assessed.

### Statistical Analysis

Cox proportional hazards regression models were used to estimate hazard ratios (HRs) of death and 95% CIs associated with mean positive affect and positive affect instability. Checking Schoenfeld residuals indicated that proportional hazards assumptions were met in the Cox regression models. Five models were tested sequentially to determine whether associations of positive affect with mortality were attenuated by different types of potential confounders. Model 1 was adjusted for age and sex. Demographic factors (ie, race/ethnicity, social class, education, marital status, and employment status) were added in model 2. Model 3 adjusted for baseline illness, and model 4 added health behaviors (ie, smoking, physical activity, and alcohol intake). Mean negative affect and negative affect instability were included in model 5.

Three sensitivity analyses were conducted. First, we stratified the sample by age (≤65 vs >65 years) to check whether associations varied with age. Second, we addressed the issue of whether reverse causation was relevant, with people nearing the end of life showing terminal decline in positive affect. We therefore restricted the analyses to individuals who survived at least 24 months after EMA assessment. Third, to deal with missing data, we used multiple imputation using chained equations in the prediction model to generate 20 imputed data sets (each of 4027 individuals), and repeated the Cox regression analysis. Analyses were performed using SPSS version 26 (IBM) and STATA version 15 (StataCorp). *P* values were 2-tailed, and statistical significance was set at *P* < .05. Data were analyzed from September 2019 to April 2020.

## Results

There were 4353 individuals in wave 2 of the English Longitudinal Study of Ageing with measures of affect instability who were tracked for mortality. After omitting 326 individuals with cancer at baseline and 193 individuals with missing data on any covariates, the sample size was 3834 individuals (mean [SD] age at baseline, 64.0 [7.4] years; 2082 [54.3%] women), of whom 767 (20.0%) died during the follow-up period. All-cause mortality was monitored up to March 2018 (mean [SD] follow-up, 12.25 [2.60] years) and analyzed with Cox proportional hazards regression. Table 1 summarizes the characteristics of participants and levels of covariates included in the regression models. Positive and negative affects ranged from 1 to 4 (the full range available). The MSSDs ranged from 0 to 4 for positive affect and 0 to 3.55 for negative affect.

**Table 1.** Characteristics of the Study Sample

Variable	No. (%)
Age, mean (SD), y	64.0 (7.4)
Sex	
Men	1752 (45.7)
Women	2082 (54.3)
White race/ethnicity	52 (1.4)
Education^a^	
Lower	1179 (30.8)
Intermediate	1569 (40.9)
Higher	1086 (28.3)
Occupational class^b^	
Routine	1464 (38.2)
Intermediate	948 (24.7)
Managerial	1422 (37.1)
Paid employment	1294 (33.8)
Married or cohabiting	2852 (74.4)
Current smoker	443 (11.6)
Daily alcohol consumption	1148 (29.9)
Positive affect, mean (SD)	
Overall^c^	1.87 (0.42)
Instability^d^	0.137 (0.252)
Negative affect, mean (SD)	
Overall^c^	1.16 (0.31)
Instability^d^	0.068 (0.202)
Illness at baseline	
Coronary heart disease	111 (2.9)
Diabetes	260 (6.8)
Chronic lung disease	165 (4.3)
Stroke	64 (1.7)
Other limiting longstanding illness	1200 (30.4)
Weekly MVPA	3217 (83.9)

Abbreviation: MVPA, moderate to vigorous physical activity.

^a^ Based on the highest qualifications obtained. Low indicates no qualifications; intermediate, junior and senior high school; and higher, college.

^b^ Based on the National Statistics Socio-economic Classification.

^c^ Computed based on self-rating of mood from 4 time points during the course of 1 day: soon after waking, 30 minutes after waking, at 7:00 PM, and at bedtime, with greater scores indicating greater affect. Positive affect was computed by calculating the mean of 3 positive states (ie, happy, excited, and content), and negative affect by calculating the mean of 3 negative states (ie, worried, anxious, and fearful).

^d^ Calculated using mean squared successive difference to capture the extent of individuals’ change from 1 time point to the next, with higher scores corresponding to higher variability.

We analyzed 5 models, as shown in Table 2. Model 1 included mean positive affect and positive affect instability together with age and sex. Higher positive affect instability was associated with greater mortality (hazard ratio [HR], 1.24; 95% CI, 1.07-1.44; *P* = .004), while higher mean positive affect was associated with lower mortality (HR, 0.83; 95% CI, 0.70-0.99; *P* = .03). These associations remained significant in model 2 when race/ethnicity, educational attainment, social class, marital status, and paid employment were included as covariates. In model 3, the association of greater positive affect instability with mortality was maintained, but there was no protective association for mean positive affect. The addition of health behaviors in model 4 did not alter the association of positive affect instability and mortality. Finally, in model 5, the independent association of greater positive affect instability with mortality was unchanged (HR, 1.25; 95% CI, 1.04-1.49; *P* = .02). The full results of model 5 are presented in Table 3. In addition to positive affect instability, older age, male sex, being unmarried, having a diagnosed or limiting long-standing illness at baseline, smoking, and being physically inactive were independently associated with mortality. Neither mean negative affect nor negative affect instability were significantly associated with mortality in the final model.

**Table 2.** Associations of Positive Affect Measures With Mortality

Model (covariates)	Adjusted HR (95% CI)	*P* value
1 (Age, sex)		
Mean positive affect	0.83 (0.70-0.99)	.04
Positive affect instability	1.24 (1.07-1.44)	.004
2 (Age, sex, demographic factors)		
Mean positive affect	0.84 (0.71-0.99)	.04
Positive affect instability	1.24 (1.07-1.44)	.005
3 (Age, sex, demographic factors, baseline illness)		
Mean positive affect	0.90 (0.76-1.07)	.22
Positive affect instability	1.24 (1.04-1.46)	.01
4 (Age, sex, demographic factors, baseline illness, smoking, alcohol intake, physical activity)		
Mean positive affect	0.93 (0.79-1.11)	.43
Positive affect instability	1.24 (1.04-1.47)	.02
5 (Age, sex, demographic factors, baseline illness, smoking, alcohol intake, physical activity, mean negative affect, negative affect instability)		
Mean positive affect	0.92 (0.77-1.10)	.36
Positive affect instability	1.25 (1.04-1.49)	.02

Abbreviation: HR, hazard ratio.

**Table 3.** Factors Associated With Mortality in the Fully Adjusted Model

Factor	Adjusted HR (95% CI)	*P* value
Positive affect		
Instability	1.25 (1.04-1.49)	.02
Mean	0.92 (0.77-1.10)	.36
Age	1.12 (1.10-1.13)	<.001
Female sex	0.57 (0.49-0.66)	<.001
Education^a^		
Lower	1 [Reference]	.15
Intermediate	0.85 (0.67-1.06)	.04
Higher	0.84 (0.71-0.99)	
Occupational class^b^		
Routine	1 [Reference]	
Intermediate	0.98 (0.81-1.17)	.80
Managerial	0.93 (0.77-1.13)	.47
Not in paid employment	1.14 (0.89-1.46)	.30
Unmarried	1.18 (1.01-1.38)	.048
Illness at baseline		
None	1 [Reference]	
Diabetes	1.25 (0.99-1.67)	.05
Coronary heart disease	1.19 (0.87-1.61)	.28
Stroke	1.54 (1.07-2.21)	.02
Chronic lung disease	1.93 (1.51-2.46)	<.001
Limiting long-standing illness	1.45 (1.24-1.70)	<.001
Smoking	2.09 (1.72-2.54)	<.001
Daily alcohol consumption	1.02 (0.86-1.19)	.86
Weekly MVPA	1.39 (1.17-1.65)	<.001
Negative affect		
Instability	1.11 (0.77-1.60)	.57
Mean	0.90 (0.68-1.17)	.40

Abbreviation: HR, hazard ratio; MVPA, moderate to vigorous physical activity.

^a^ Based on the highest qualifications obtained. Low indicates no qualifications; intermediate, junior and senior high school; and higher, college.

^b^ Based on the National Statistics Socio-economic Classification.

### Sensitivity Analysis

We conducted 3 sensitivity analyses. First, we considered whether the pattern of results varied by age. In the models including all covariates, associations of positive affect instability with mortality were similar in the younger (HR, 1.22; 95% CI, 1.01-1.46) and older age groups (HR, 1.37; 95% CI, 0.72-2.61). Second, we omitted individuals who died within 24 months of the positive affect assessment as a guard against reverse causation. The sample size was reduced to 3785 individuals, with 719 deaths between 24 months and the census date. The association of positive affect instability with mortality was unchanged (HR, 1.24; 95% CI, 1.04-1.41; *P* = .02). Finally, we repeated the analysis following multiple imputation for missing data on covariates, so the sample size was increased to 4027 individuals. The association of mortality with positive affect instability was similar to that in the unimputed data set (HR, 1.30; 95% CI, 1.26-1.35), and the protective association with mean positive affect was significant in the fully adjusted model (HR, 0.93; 95% CI, 0.90-0.97).

## Discussion

This survey study tested the association of positive affective instability with mortality. Our analyses indicate that temporal fluctuations in positive affect assessed during a single day were associated with mortality over a 12-year follow-up, with high positive affect instability associated with greater mortality in a national sample of older adults in England. Notably, the association was independent of mean levels of positive affect and negative affect, suggesting that the association of positive affect instability with mortality risk may not simply be due to mean levels of affective well-being. Findings for all-cause mortality were replicated in analyses controlling for sociodemographic factors, health at baseline, major health behaviors, and instability in negative affective states.

This study advances current research on positive affect and health in several ways. First, it shows that short-term instability in positive affective states was associated with reduced longevity. Previous research has documented associations of mean levels of positive affect with survival,^11,15,16^ but not temporal fluctuations, or ups and downs, in positive affect. Our finding that positive affect instability was associated with mortality lends further evidence that variability and temporal dependency are 2 fundamental properties of affect dynamics that are characteristic of poor health.^27,35^ In general, these findings are important because they provide evidence for both enduring and fragile forms of affective phenomena occurring in people’s everyday lives.^17^ Second, we adjusted for a wide range of potential demographic, health, affective, and behavioral factors that may confound the association of positive affect instability with mortality. A critical methodological issue in studies of psychological well-being and mortality is whether associations are independent of negative affective states.^13,30^ In our analysis, the association of positive affect instability with mortality remained significant after adjusting for EMA measures of mean negative affect and negative affect instability, suggesting that time-ordered oscillations in positive affect convey distinct information about people’s everyday lives, especially regarding mortality risk. Third, associations did not differ by age, indicating that the association of positive affect instability with survival was not restricted to older adults.

### Limitations

This study has some limitations. This is an observational study, so causal conclusions cannot be drawn. It is possible that the pattern of deaths resulted from reverse causality. However, associations were maintained when controlling for self-rated health and when excluding deaths that occurred within 2 years of the EMA assessment, arguing against the possibility that subclinical health issues or terminal decline processes led to greater positive affect instability and early mortality. A limitation of this analysis was that EMA was carried out over a single day, and only 4 assessment ratings were completed. These data were collected in the context of a much larger study of social, economic, psychological, and medical aspects of aging, and there was no opportunity to extend the assessments. Additional ratings across several days may give a more reliable estimate of how people feel in everyday life and capture more ecologically valid associations with mortality. Additionally, the analyses were based on all-cause mortality, and additional research is required to explore which specific causes of death may be associated with positive affect instability.

## Conclusions

Short-term fluctuations in positive affect have been associated with psychological maladjustment,^20,21^ as well as health behaviors^22,23^ and biological correlates^24,25,26^ that may increase risk of adverse physical health outcomes. The findings of this survey study provide further evidence that although sustained positive well-being may promote longevity,^36^ unstable feelings of positive affect may increase mortality risk. These findings highlight the need to obtain more insight into the processes at play in the association of positive affect instability with mortality. Lifestyle factors (eg, physical activity and healthy diet)^30^ and biological processes (eg, cortisol and heart rate)^3^ have been associated with health and well-being and will need to be investigated as possible mechanisms in future studies. Such research will provide information relevant to interventions aimed at improving the lives of older adults.
